# Supplementary material for: Recombinase‐mediated integration of a multigene cassette in rice leads to stable expression and inheritance of the stacked locus
Source: Plant Direct. 2020 Jul 6;4(7):e00236. doi: 10.1002/pld3.236 (PMC7391932; doi:10.1002/pld3.236)
Supplement: Supplementary file 1 — Fig S1‐S4 [file PLD3-4-e00236-s001.pptx]

## Slide 1
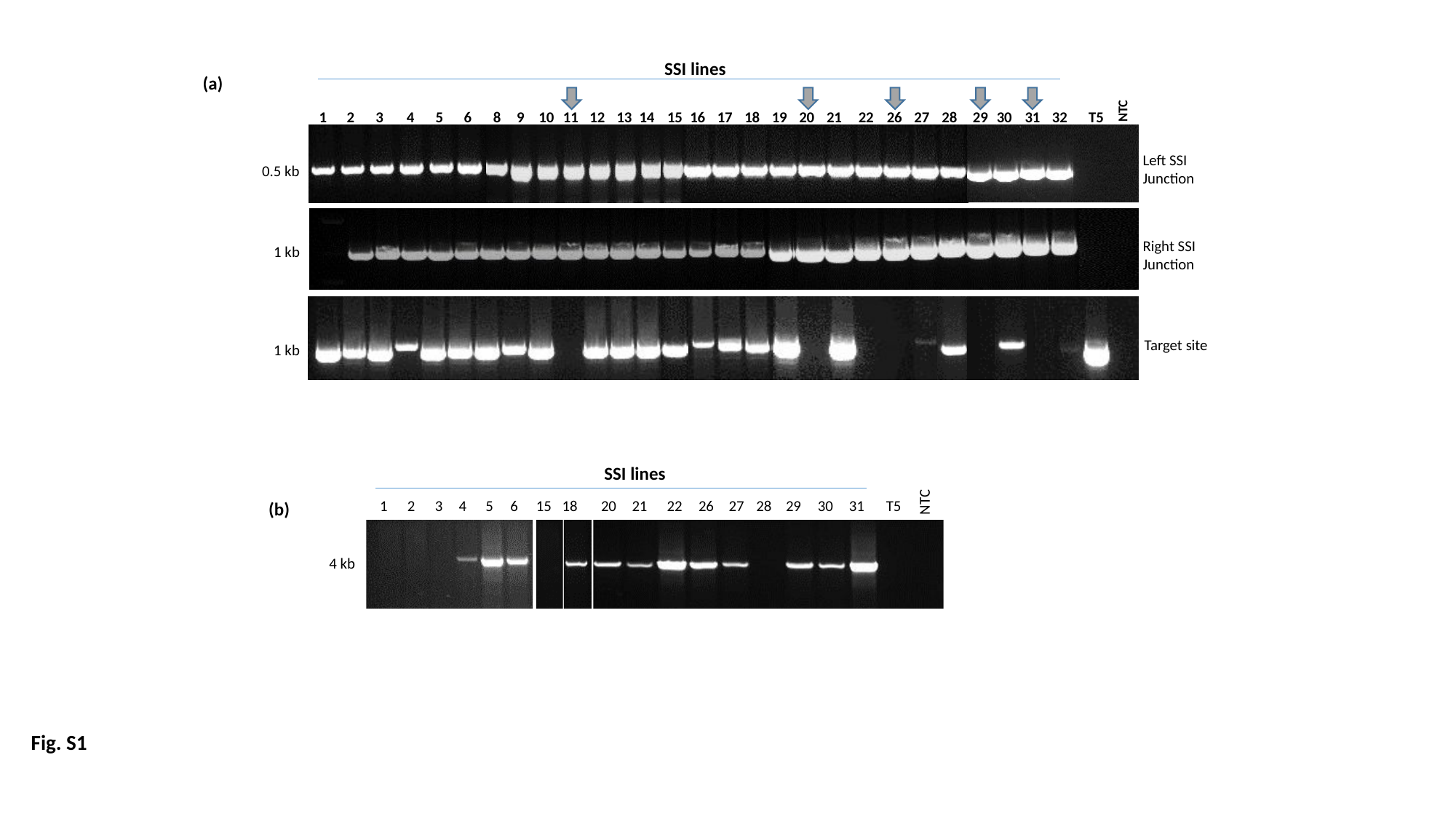

SSI lines
(a)
NTC
1
2
3
4
5
6
8
9
10
11
12
13
14
15
16
17
18
19
20
21
22
26
27
28
29
30
31
32
T5
Left SSI Junction
0.5 kb
Right SSI Junction
1 kb
Target site
1 kb
SSI lines
NTC
15
18
1
2
3
4
5
6
21
22
26
27
28
29
30
31
T5
20
4 kb
(b)
Fig. S1

## Slide 2
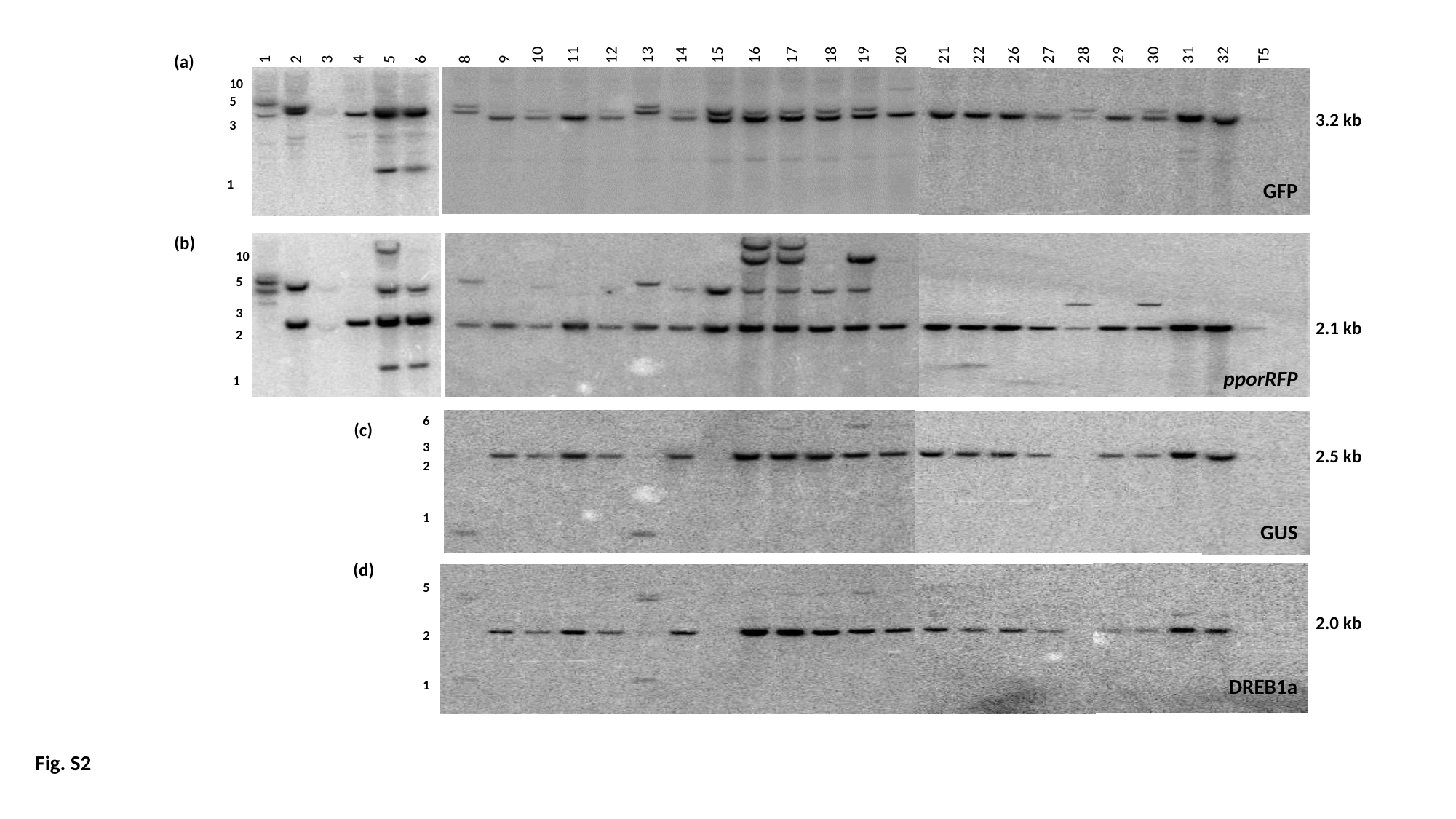

3
6
8
9
10
11
12
13
14
15
16
17
18
19
20
21
22
26
27
28
29
30
31
32
T5
1
2
4
5
(a)
10
5
3.2 kb
3
1
GFP
(b)
10
5
3
2.1 kb
2
pporRFP
1
6
(c)
3
2.5 kb
2
1
GUS
(d)
5
2.0 kb
2
DREB1a
1
Fig. S2

## Slide 3
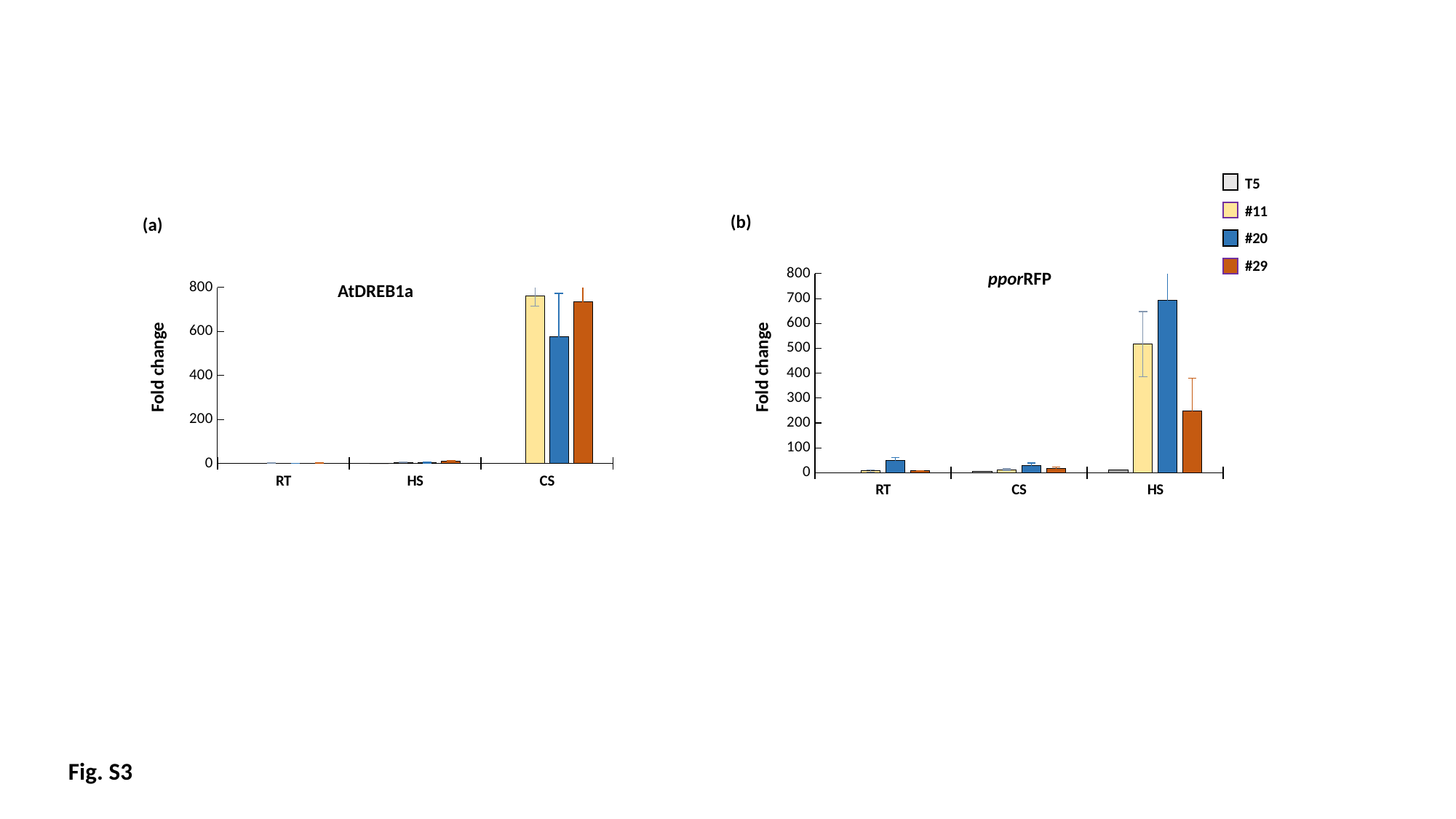

T5
#11
#20
#29
(b)
(a)
pporRFP
### Chart
| Category | T5 | 11 | 20 | 29 |
|---|---|---|---|---|
| RT | 1.0 | 7.113755638298757 | 48.41265098807038 | 7.506249464406357 |
| CS | 4.53421645466757 | 12.664325839224995 | 27.802602251516888 | 16.60797506082733 |
| HS | 12.058663533669286 | 516.7018974786856 | 693.1352745384056 | 246.95011190221263 |
### Chart
| Category | T5 | 11 | 20 | 29 |
|---|---|---|---|---|
| RT | 1.0 | 0.3300521809793706 | 0.37204503305504566 | 1.2687603617410113 |
| HS | 0.17161 | 4.88256136211713 | 4.292881676111559 | 9.32366125193763 |
| CS | 1.1728812110562252 | 760.8425065806246 | 577.0328735804824 | 735.3607128217102 |AtDREB1a
Fold change
Fold change
Fig. S3

## Slide 4
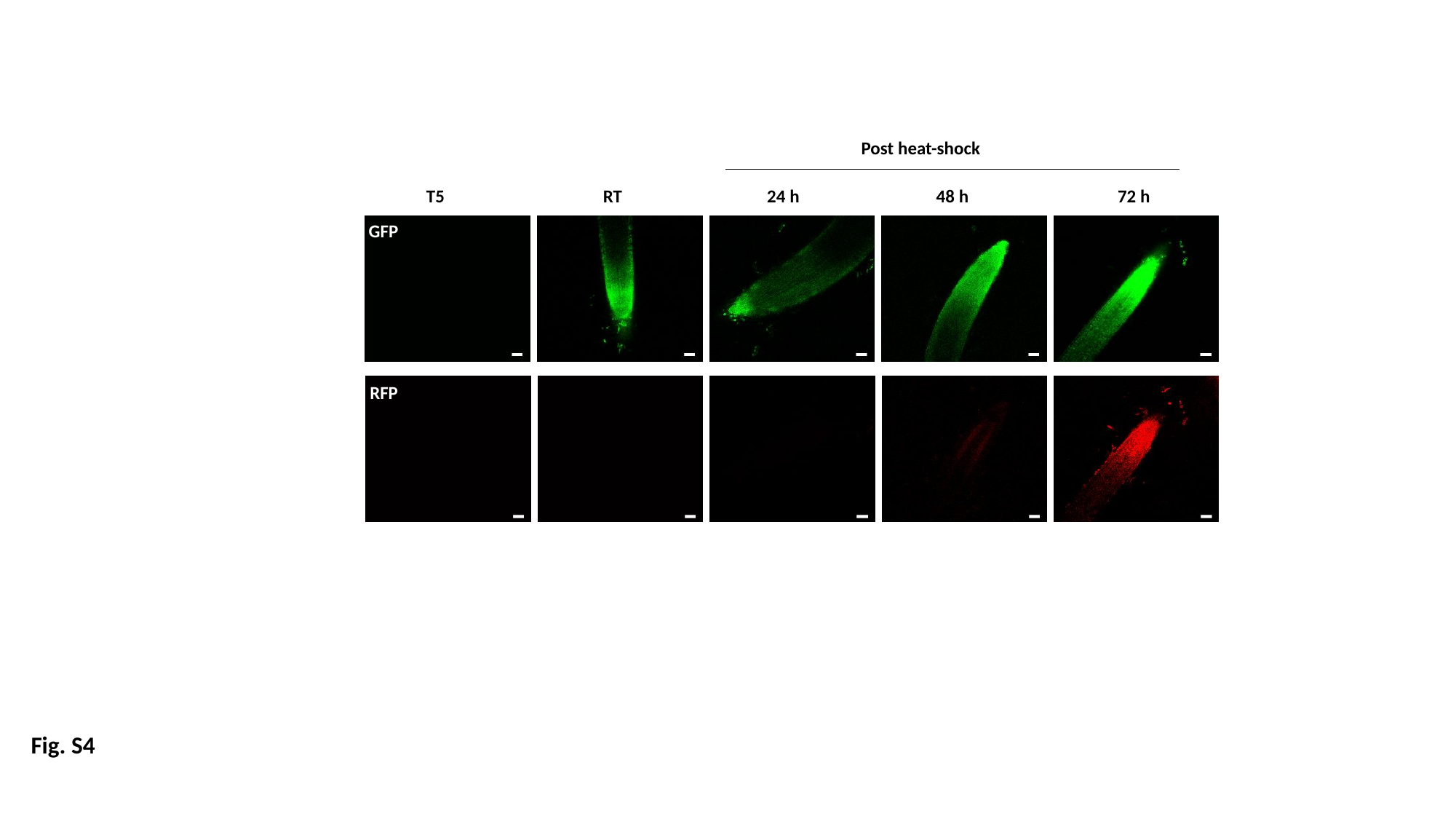

Post heat-shock
T5
RT
24 h
48 h
72 h
GFP
RFP
GFP
Fig. S4
